# Comparative analysis of the effects of aztreonam combined with four β-lactamase inhibitors against carbapenem-resistant *Enterobacterales*

**DOI:** 10.1128/spectrum.01408-25

**Published:** 2026-01-30

**Authors:** Qing Meng, Chao Xu, Wujiao Li, Kunling Shen, Xiuhui Huang, Xiaoying Fu, Lintao Zhou, Yunsheng Chen, Fupin Hu, Heping Wang

**Affiliations:** 1 Department of Clinical Microbiology Laboratory, Shenzhen Children’s Hospital, Shenzhen, Guangdong, China; 2 First Clinical Medical College, Ningxia Medical University 105002, Yinchuan, Ningxia, China; 3 Department of Respiratory Diseases, Shenzhen Children’s Hospital, Shenzhen, Guangdong, China; 4 Department of Respiratory Medicine, Beijing Children's Hospital, Capital Medical University, National Center for Children's Health 117984 , Beijing, China; 5 Institute of Antibiotics, Huashan Hospital, Fudan University 12478, Shanghai, China; 6 Key Laboratory of Clinical Pharmacology of Antibiotics, Ministry of Health, Shanghai, China; bioMerieux Inc, Eugene, Oregon, USA

**Keywords:** carbapenem-resistant *Enterobacterales*, aztreonam/avibactam, metallo-β-lactamase, clavulanic acid

## Abstract

Carbapenem-resistant *Enterobacterales* (CRE) pose a global public health threat due to multidrug resistance and treatment failure. This study evaluated the effects of aztreonam combined with four β-lactamase inhibitors (avibactam, clavulanic acid, tazobactam, and sulbactam) on 77 CRE isolates from Shenzhen Children’s Hospital. Among these isolates, 57.1% harbored metallo-β-lactamases (MBL) genes, and most co-harbored extended-spectrum β-lactamase (ESBL)/AmpC genes. Using microbroth dilution, checkerboard assays, and time‒kill curves, aztreonam/clavulanic acid showed potent synergy against MBL^+^/AmpC^−^ strains (26/27), with MIC50/MIC90 values (≤0.12/1 µg/mL) comparable to those of aztreonam/avibactam (≤0.12/0.5 µg/mL). Aztreonam/tazobactam exhibited lower synergism, while aztreonam/sulbactam had indifferent effects. Time‒kill assays confirmed the similar bactericidal activity between aztreonam/clavulanic acid and aztreonam/avibactam against MBL^+^/ESBL^+^ isolates. Although aztreonam/avibactam maintains broad efficacy, these *in vitro* studies suggest that the aztreonam/clavulanic acid combination regimen should be further explored as a potential and cost-effective alternative treatment option for MBL^+^/AmpC^−^ CRE, which is expected to reduce the economic burden and delay avibactam resistance. These findings support tailored β-lactamase inhibitor selection on the basis of resistance mechanisms.

**IMPORTANCE:**

Carbapenem-resistant *Enterobacterales* (CRE) pose significant risks for pediatric infections, with few safe and affordable therapeutic options available. This study focuses on a practical issue in current clinical research: aztreonam combined with clavulanic acid shows certain *in vitro* activity against CRE strains that carry metallo-β-lactamases gene but lack AmpC enzymes gene—a common profile among pediatric CRE isolates.

Compared to the costly aztreonam/avibactam, this combination presents a potential low-cost candidate, which may help alleviate economic burdens on healthcare systems and patients if further validated. Additionally, the findings provide reference for reducing over-reliance on newer antibiotics like avibactam, supporting efforts to delay antimicrobial resistance. This work may be of practical use for regions with limited access to high-cost antimicrobials.

## INTRODUCTION

Antimicrobial resistance has become a public health issue of global concern (1). The Bacterial Priority Pathogens List updated by the WHO in 2024 listed carbapenem-resistant *Enterobacterales* (CRE) as a critical group (2). Carbapenems were once the last line of defense for the treatment of infections caused by *Enterobacterales*. Owing to the widespread use of carbapenems in clinical practice, the detection rate of CRE has increased annually. According to a report from the China Antimicrobial Surveillance Network (CHINET), the resistance rate of *Klebsiella pneumoniae* to imipenem increased from 3% in 2005 to 20.9% in 2017 in China (3).

The mortality rate of patients with CRE bloodstream infection has been reported to be as high as 32.9% (4). Previous reports have shown that the traditional antibiotics with low resistance rates in CRE include amikacin, polymyxins, and tigecycline (5, 6). In 2023, the Clinical and Laboratory Standards Institute (CLSI) lowered the breakpoint for amikacin for *Enterobacterales* (7), and amikacin was recommended to be used in combination with other antibiotics for infections other than uncomplicated cystitis and pyelonephritis. Polymyxins have severe nephrotoxicity and poor clinical efficacy, and tigecycline has a large volume of distribution, which limits the ability of free drug concentrations in the blood to reach therapeutic levels for treating bacteremia (8, 9). In contrast, the new β-lactamase inhibitor combination has good clinical application value. It has been reported that ceftazidime/avibactam has a higher clinical treatment success rate to CRE than colistin (10, 11), meropenem/vaborbactam was associated with lower 28-day all-cause mortality compared with best available therapy (monotherapy/combination therapy with polymyxins, carbapenems, aminoglycosides, colistin, and tigecycline; or ceftazidime/avibactam; or piperacillin/tazobactam) in treating CRE infections (12), and aztreonam/avibactam has good antibacterial activity against metallo-β-lactamases (MBLs) producing CRE strains and still has antibacterial activity against ≥98.9% of CRE strains resistant to ceftazidime/avibactam, meropenem/vaborbactam, and imipenem/relebactam (13).

Understanding the resistance mechanism of CRE is highly important for the treatment of CRE infection and the development of new antimicrobial agents. The resistance mechanism of CRE to β-lactams mainly involves carbapenemase production or high AmpC enzyme production combined with membrane porin deficiency or high expression of efflux pumps, and the type of carbapenemase is closely related to the choice of antimicrobial treatment regimen (8, 14). In 2019, the new β-lactamase inhibitor combination ceftazidime/avibactam was launched in mainland China and approved for use in children in 2020. Studies have shown that avibactam has good antibacterial activity against carbapenemases, including KPC-2 and OXA-48-like carbapenemases, but is ineffective against some *Klebsiella pneumoniae* carbapenemase (KPC) variants and MBLs (6, 15). Previous studies have shown that the production of MBLs in carbapenemase-producing *Enterobacterales* (CPE) isolates from children is >50% in China (6, 16). Since MBLs cannot hydrolyze aztreonam and since avibactam can inhibit the extended-spectrum β-lactamases (ESBLs) and AmpC enzymes that are produced simultaneously by most CRE isolates (8), the Infectious Diseases Society of America (IDSA) guidelines recommend that other than urinary tract infections, the treatment of infections caused by MBL-producing CRE isolates should first employ aztreonam combined with ceftazidime/avibactam or cefiderocol monotherapy (14). However, because cefiderocol is not yet available on the market in mainland China, only aztreonam combined with ceftazidime/avibactam can be used as the first choice for treatment. In addition, reports have shown that cirA deleterious alteration, PBP3 insertion, and NDM production can lead to cefiderocol resistance, but aztreonam/avibactam can still maintain sensitivity (17).

As early as 2018, it was reported that aztreonam combined with clavulanic acid was a good indicator treatment for detecting ESBL in MBL-producing isolates (18). However, at that time, this treatment was reported only as a means of detecting ESBL resistance mechanisms. In addition, since *Stenotrophomonas maltophilia* naturally produces MBL, some studies have reported that aztreonam combined with clavulanic acid or avibactam also has good antibacterial activity against *S. maltophilia* (19–21), with the earliest report published in 1991 (21). There have also been similar reports on MBL-producing *Enterobacterales* or *Pseudomonas aeruginosa* (20, 22–25), but some studies have used only the Etest strip superposition method or time‒kill curves, and the number of test isolates was relatively small (20, 22–25). The purpose of our study was to compare the antibacterial activity of aztreonam combined with avibactam, clavulanic acid, sulbactam, or tazobactam against CRE from children that produce different β-lactamases and to find a more economical combination treatment option than aztreonam combined with avibactam.

## MATERIALS AND METHODS

### Isolate collection

Aztreonam-resistant CREs from clinical departments at Shenzhen Children’s Hospital between 2014 and 2021 were collected, and duplicate isolates from the same child were excluded. The VITEK MS MALDI-TOF and VITEK 2-Compact platforms from BioMérieux, France, were used for isolate identification. CRE was defined as *Enterobacterales* resistant to any carbapenem, and *Proteus* spp., *Morganella* spp., and *Providencia* spp. were defined as resistant to other carbapenems in addition to imipenem (26).

### Antimicrobial susceptibility tests

Antimicrobial susceptibility tests were performed via the microbroth dilution method recommended by the CLSI to determine the minimum inhibitory concentrations (MICs) of various antibiotics against CRE (27). The microbroth dilution susceptibility plates were purchased from Thermo Fisher Scientific; the avibactam standard was purchased from Dalian Meilun Company; and the aztreonam, clavulanic acid, sulbactam, and tazobactam standards were purchased from the China Food and Drug Administration (FDA). The corresponding solvents and diluents for the antimicrobial drug standards were purchased from Sinopharm Reagent Company. The antimicrobial susceptibility test results were interpreted according to the CLSI standard (7), and the US FDA breakpoint standard was used to interpret the results of the tigecycline and aztreonam/avibactam antimicrobial susceptibility test (28, 29); the cefoperazone‒sulbactam results were interpreted according to the breakpoint standard for cefoperazone (30).

Microbroth dilution checkerboard and time-kill assays were used to evaluate the synergistic effects of aztreonam combined with avibactam, clavulanic acid, sulbactam, or tazobactam on aztreonam-resistant CRE; for the test methods, refer to the Clinical Microbiology Procedures handbook (31). Both the microbroth dilution checkerboard and time-kill assays were performed in cation-adjusted Mueller–Hinton broth under ambient air conditions. For the checkerboard assay, aztreonam was twofold serially diluted horizontally across 12 × 12 microtiter plates to obtain final concentrations ranging from 0.12 to 128 μg/mL. Vertically, avibactam, clavulanic acid, sulbactam, or tazobactam was likewise diluted to the same concentration range. The synergistic effects between aztreonam and each β-lactamase inhibitor were evaluated in triplicate to ensure reproducibility. For the time‒kill assay, bacterial growth was assessed in triplicate under the following conditions: drug-free growth control, aztreonam alone, and aztreonam combined with 4 μg/mL avibactam, clavulanic acid, sulbactam, or tazobactam. After inoculation, samples were collected at 0, 2, 4, 8, 10, and 24 h, plated on blood agar, and colonies were counted to determine viable bacterial concentrations. The mean values from three independent experiments were used for the final analysis.

Three different concentrations of aztreonam and the β-lactamase inhibitor were used: the concentration ratio of aztreonam to the β-lactamase inhibitor was 2:1, and the concentrations of the β-lactamase inhibitor were fixed at 4 µg/mL and 2 µg/mL to test the MIC distributions of the test strains.

### Quality control isolates

*Enterococcus faecalis* ATCC 29212, *Staphylococcus aureus* ATCC 29213, *Escherichia coli* ATCC 25922 and ATCC 35218, *P. aeruginosa* ATCC 27853, and *K. pneumoniae* ATCC 700603 were used as quality control isolates. The corresponding quality control strains were used to conduct quality control tests every time an antimicrobial susceptibility test was conducted to ensure that the results of the antimicrobial susceptibility test were reliable.

### Whole-genome sequencing to detect β-lactamase genes and multilocus sequence typing

Genomic DNA was extracted from each isolate via a TIANamp Bacteria DNA Kit (Beijing Tiangen Biochemical Technology Co., Ltd.). The bacterial genomes were sequenced on the Illumina NovaSeq 6000 platform via 150 bp PE reads, and fastp was performed to remove low-quality reads. The genomes were *de novo* assembled with the SPAdes Genome Assembler (version: 3.15.5). A Resistance Gene Identifier (version: 6.0.0) was used to analyze common β-lactamase genes (carbapenemase genes: NDM, IMP, VIM, KPC, and OXA-48-like; narrow-spectrum β-lactamase (NSBL) genes: TEM, SHV, and OXA-1; ESBL genes: TEM, SHV, OXA-10, and CTX-M; AmpC β-lactamase genes: DHA, CMY, and ACT), and the (32) multilocus sequence typing (MLST) analysis was performed with MLST (https://github.com/tseemann/mlst, version: 2.23.0).

### Statistical analysis

Statistical analysis of the antimicrobial susceptibility results was performed via WHONET 5.6 software. GraphPad Prism 9.5.1 software was used to generate visuals of the data. The synergistic effect of aztreonam combined with the four β-lactamase inhibitors was determined by calculating the fractional inhibitory concentration (FIC) index, FIC = (MIC of aztreonam in combination/MIC of aztreonam alone) + (MIC of β-lactamase inhibitor in combination/MIC of β-lactamase inhibitor alone), with an FIC ≤ 0.5 indicating synergism, an FIC > 0.5 to −1 indicating addition, an FIC > 1—2 to −2 indicating indifference, and an FIC > 2 indicating antagonism (33).

## RESULTS

### Bacterial distribution and antimicrobial susceptibility results

To determine the combined efficacy of aztreonam with different enzyme inhibitors, we selected CREs that were resistant to aztreonam and tested the resistance of these strains via the microbroth dilution method. From 2014 to 2021, a total of 77 aztreonam-resistant CREs, including *K. pneumoniae* (*n* = 42, 54.5%), *E. coli* (*n* = 15, 19.5%), *Klebsiella aerogenes* (*n* = 10, 13%), *Enterobacter cloacae* (*n* = 8, 10.4%), and one isolate each of *Citrobacter freundii* and *Proteus vulgaris*, were isolated at Shenzhen Children’s Hospital. The resistance rates of these CREs to all the tested antibiotics except amikacin, polymyxin B, and tigecycline were >50%. The MIC50 and MIC90 values of amoxicillin/clavulanic acid, cefoperazone/sulbactam, ceftazidime/avibactam, piperacillin/tazobactam, cefazolin, cefuroxime, ceftazidime, ceftriaxone, cefepime, and trimethoprim/sulfamethoxazole were >32 µg/mL. The resistance rate to ceftazidime/avibactam was 55.8%. The MIC50 and MIC90 values of aztreonam/avibactam were 0.25 µg/mL and 2 µg/mL, respectively, and both values were low. *K. pneumoniae* and *E. coli* had higher resistance rates to cefepime, meropenem, amikacin, ciprofloxacin, and levofloxacin than did the *E. cloacae* complex and *K. aerogenes*, but they had lower resistance rates to imipenem, trimethoprim/sulfamethoxazole, and polymyxin B (Table 1).

**TABLE 1:** Antimicrobial susceptibility test results of 77 aztreonam-resistant CRE

Antibiotic	Aztreonam-resistant CRE (*n* = 77)					*K. pneumoniae* and *E. coli* (*n* = 57)					*E. cloacae* complex and *K. aerogenes* (*n* = 18)				
	%R	%S	MIC50 (µg/mL)	MIC90 (µg/mL)	MIC range (µg/mL)	%R	%S	MIC50 (µg/mL)	MIC90 (µg/mL)	MIC range (µg/mL)	%R	%S	MIC50 (µg/mL)	MIC90 (µg/mL)	MIC range (µg/mL)
Aztreonam/avibactam	0	97.4	0.25	2	≤0.12–8	0	98.2	0.25	1	≤0.12–8	0	94.4	0.5	4	≤0.12–8
Amoxicillin/clavulanic acid	97.4	1.3	64	>128	8 to >128	96.5	1.8	64	>128	8 to >128	100	0	64	128	64 to >128
Cefoperazone/sulbactam	96.1	2.6	>128	>128	16 to >128	100	0	>128	>128	64 to >128	88.9	5.6	>128	>128	16 to >128
Ceftazidime/avibactam	55.8	44.2	>64	>64	≤0.03 to >64	56.1	43.9	64	>64	≤0.03 to >64	55.6	44.4	>64	>64	0.25 to >64
Piperacillin/tazobactam	88.3	7.8	>256	>256	≤2 to >256	91.2	8.8	>256	>256	8 to >256	83.3	0	>256	>256	32 to >256
Cefazolin	100	0	>32	>32	>32	100	0	>32	>32	>32	100	0	>32	>32	>32
Cefuroxime	100	0	>32	>32	>32	100	0	>32	>32	>32	100	0	>32	>32	>32
Ceftazidime	94.8	0	>32	>32	8 to >32	94.7	0	>32	>32	8 to >32	100	0	>32	>32	32 to >32
Ceftriaxone	100	0	>32	>32	>32	100	0	>32	>32	>32	100	0	>32	>32	>32
Cefepime	94.8	3.9	128	>128	1 to >128	100	0	>128	>128	16 to >128	77.8	16.7	32	>128	1 to >128
Aztreonam	100	0	>128	>128	16 to >128	100	0	>128	>128	16 to >128	100	0	32	>128	16 to >128
Imipenem	62.3	28.6	8	32	≤0.06 to>128	59.6	29.8	8	32	≤0.06 to >128	66.7	27.8	16	64	0.25 to >128
Meropenem	71.4	15.6	16	>64	0.06 to >64	75.4	8.8	16	>64	0.25 to >64	61.1	38.9	8	64	0.06–64
Amikacin	7.8	90.9	≤1	16	≤1 to >128	10.5	87.7	2	>128	≤1 to >128	0	100	≤1	2	≤1–4
Ciprofloxacin	68.8	27.3	2	>8	≤0.06 to >8	73.7	22.8	8	>8	≤0.06 to >8	61.1	33.3	1	8	≤0.06–8
Levofloxacin	59.7	33.8	2	>16	≤0.125 to >16	64.9	28.1	8	>16	≤0.125 to >16	50	44.4	1	2	≤0.125–2
Trimethoprim/sulfamethoxazole	61	39	>32	>32	≤0.25 to >32	59.6	40.4	>32	>32	≤0.25 to >32	72.2	27.8	>32	>32	≤0.25 to >32
Polymyxin B	3.9	0	0.5	1	0.5 to >16	0	0	0.5	1	0.5-2	11.1	0	0.5	>16	0.5 to >16
Tigecycline	0	100	0.25	1	≤0.06–2	0	100	0.25	1	≤0.06 to 2	0	100	0.5	1	0.25-2

### Distribution of the β-lactamase gene and results of the checkerboard dilution method

To investigate the synergistic effects of aztreonam combined with different enzyme inhibitors on CRE strains with various resistance mechanisms, we identified the resistance mechanisms of these strains through whole-genome sequencing and tested the combined effects via the checkerboard dilution method. Among the 77 aztreonam-resistant CRE isolates, 57.1% carried an MBL gene, and the KPC gene (11.9%) was detected in only *K. pneumoniae*. The rates of ESBL gene detection in *K. pneumoniae* and *E. coli* were 90.5% and 93.3%, respectively, and these values were higher than those in *K. aerogenes* (30%) and the *E. cloacae* complex (12.5%). The detection rates of the AmpC enzyme-encoding genes in *K. pneumoniae* and *E. coli* were 11.9% and 26.7%, respectively, which were lower than those in *K. aerogenes* (100%) and the *E. cloacae* complex (87.5%). The results of the checkerboard dilution method revealed that for all 77 CRE isolates, aztreonam combined with avibactam had synergistic effects. The most common effects of aztreonam combined with clavulanic acid, sulbactam, or tazobactam were synergism (50.6%), indifference (68.8%), and synergism (40.2%). The rates of synergistic effects of aztreonam combined with clavulanic acid on *K. pneumoniae* and *E. coli* were 52.3% and 80%, respectively, and these rates were greater than those of *K. aerogenes* (10%) and the *E. cloacae* complex (25%). The rates of antagonistic effects on *K. pneumoniae* and *E. coli* were 2.3% and 0%, respectively, and these rates were lower than those of *K. aerogenes* (30%) and the *E. cloacae* complex (12.5%). The synergistic effect of aztreonam combined with tazobactam on various isolates was similar to that of aztreonam combined with clavulanic acid, but the rate of synergistic effects was slightly lower. In the aztreonam combined with sulbactam test group, the treatment effects on *K. pneumoniae*, *E. coli*, *K. aerogenes*, and the *E. cloacae* complex were mostly indifferent. The distribution of β-lactamase genes in each isolate and the combined drug sensitivity results of the checkerboard dilution method are shown in Tables 2 and 3 and Table S1.

**TABLE 2:** Distribution of β-lactamase genes and checkerboard dilution results for 77 aztreonam-resistant CRE

Isolates	β-lactamase gene (%)					Aztreonam combined with avibactam (%)				Aztreonam combined with clavulanic acid (%)				Aztreonam combined with sulbactam (%)				Aztreonam combined with tazobactam (%)			
	MBL	KPC	NSBL	ESBL	AmpC	Synergism	Addition	Indifference	Antagonism	Synergism	Addition	Indifference	Antagonism	Synergism	Addition	Indifference	Antagonism	Synergism	Addition	Indifference	Antagonism
*K. pneumoniae* (*n* = 42)	41.6	11.9	83.3	90.5	11.9	100	0	0	0	52.3	26.1	19	2.3	2.3	26.1	71.4	0	38	30.9	28.5	2.3
*E. coli* (*n* = 15)	46.7	0	53.3	93.3	26.7	100	0	0	0	80	6.6	13.3	0	13.3	33.3	53.3	0	73.3	13.3	13.3	0
*K. aerogenes* (*n* = 10)	70	0	90	30	100	100	0	0	0	10	0	60	30	0	0	90	10	0	20	50	30
*E. cloacae* complex (*n* = 8)	37.5	0	37.5	12.5	87.5	100	0	0	0	25	0	62.5	12.5	25	0	75	0	25	37.5	37.5	0
*P. vulgaris* (*n* = 1)	0	0	0	0	0	100	0	0	0	100	0	0	0	100	0	0	0	100	0	0	0
*C. freundii* (*n* = 1)	100	0	0	100	100	100	0	0	0	100	0	0	0	0	100	0	0	100	0	0	0
All (*n* = 77)	57.1	6.4	71.4	74	35.1	100	0	0	0	50.6	15.5	27.2	6.4	7.7	22	68.8	1.2	40.2	25.9	28.5	5.1

**TABLE 3:** ST typing and β-lactamase genes of 77 aztreonam-resistant CRE isolates

Strain number	Species	ST type	Carbapenemase gene			NSBL gene			ESBL gene				AmpC enzyme gene		
			NDM	IMP	KPC	TEM	SHV	OXA-1	TEM	SHV	CTX-M	OXA-10	DHA	CMY	ACT
kpn-1	*K. pneumoniae*	307		IMP-38		TEM-1					CTX-M-3				
kpn-2	*K. pneumoniae*	307		IMP-38		TEM-1				SHV-28	CTX-M-3				
kpn-3	*K. pneumoniae*	215				TEM-1	SHV-1				CTX-M-3				
kpn-4	*K. pneumoniae*	307		IMP-38		TEM-1				SHV-28	CTX-M-3				
kpn-5	*K. pneumoniae*	307		IMP-38		TEM-1					CTX-M-3				
kpn-6	*K. pneumoniae*	307		IMP-38		TEM-1				SHV-28	CTX-M-3				
kpn-7	*K. pneumoniae*	2,407				TEM-1	SHV-1				CTX-M-15				
kpn-8	*K. pneumoniae*	11			KPC-2	TEM-1				SHV-125	CTX-M-65				
kpn-9	*K. pneumoniae*	105	NDM-1			TEM-1					CTX-M-104				
kpn-11	*K. pneumoniae*	11			KPC-2	TEM-1					CTX-M-65				
kpn-12	*K. pneumoniae*	11			KPC-2	TEM-1				SHV-11	CTX-M-65				
kpn-13	*K. pneumoniae*	485	NDM-1			TEM-1				SHV-27					
kpn-14	*K. pneumoniae*	485	NDM-1			TEM-1				SHV-27	CTX-M-3				
kpn-15	*K. pneumoniae*	147	NDM-1			TEM-1				SHV-11	CTX-M-3				
kpn-17	*K. pneumoniae*	485	NDM-1			TEM-1				SHV-27	CTX-M-3				
kpn-18	*K. pneumoniae*	35				TEM-1				SHV-33	CTX-M-3				
kpn-19	*K. pneumoniae*	^*a*^–	NDM-1							SHV-12					
kpn-20	*K. pneumoniae*	5,363	NDM-1									OXA-10			
kpn-21	*K. pneumoniae*	147				TEM-1		OXA-1		SHV-11	CTX-M-3		DHA-1		
kpn-22	*K. pneumoniae*	105	NDM-1			TEM-1					CTX-M-104				
kpn-23	*K. pneumoniae*	76	NDM-1			TEM-1				SHV-12	CTX-M-14 CTX-M-15				
kpn-24	*K. pneumoniae*	355	NDM-1							SHV-12					
kpn-25	*K. pneumoniae*	309				TEM-1				SHV-11	CTX-M-15				
kpn-26	*K. pneumoniae*	36				TEM-1				SHV-53	CTX-M-15				
kpn-28	*K. pneumoniae*	48				TEM-1				SHV-11	CTX-M-3				
kpn-29	*K. pneumoniae*	1,715				TEM-1	SHV-1								
kpn-30	*K. pneumoniae*	^*a*^–	NDM-1		KPC-2	TEM-1					CTX-M-17				
kpn-31	*K. pneumoniae*	2,823	NDM-1	IMP-4						SHV-12	CTX-M-55				
kpn-32	*K. pneumoniae*	2,407	NDM-5				SHV-1				CTX-M-14		DHA-1		
kpn-33	*K. pneumoniae*	22		IMP-4			SHV-1								
kpn-34	*K. pneumoniae*	35				TEM-1				SHV-33	CTX-M-3				
kpn-35	*K. pneumoniae*	22		IMP-4			SHV-1								
kpn-36	*K. pneumoniae*	37								SHV-11	CTX-M-27				
kpn-37	*K. pneumoniae*	11			KPC-2	TEM-1		OXA-1		SHV-11					
kpn-38	*K. pneumoniae*	644	NDM-1	IMP-4		TEM-1				SHV-12					
kpn-39	*K. pneumoniae*	2,407	NDM-5				SHV-1				CTX-M-14		DHA-1		
kpn-40	*K. pneumoniae*	1,308	NDM-1							SHV-12			DHA-1		
kpn-43	*K. pneumoniae*	^*a*^–				TEM-1					CTX-M-3				
kpn-44	*K. pneumoniae*	678		IMP-4		TEM-1				SHV-41			DHA-1		
kpn-45	*K. pneumoniae*	668		IMP-4											
kpn-46	*K. pneumoniae*	17				TEM-1	SHV-1				CTX-M-3				
kpn-47	*K. pneumoniae*	273	NDM-5			TEM-1				SHV-11	CTX-M-3				
eco-1	*E. coli*	6,496				TEM-1				SHV-12		OXA-10	DHA-1	CMY-2	
eco-2	*E. coli*	354	NDM-9			TEM-1					CTX-M-17				
eco-3	*E. coli*	73	NDM-1							SHV-12	CTX-M-55				
eco-5	*E. coli*	131									CTX-M-14			CMY-132	
eco-6	*E. coli*	–^*a*^							TEM-209		CTX-M-55	OXA-10			
eco-8	*E. coli*	2,003				TEM-1					CTX-M-14 CTX-M-55				
eco-9	*E. coli*	10	NDM-1			TEM-1				SHV-12	CTX-M-14				
eco-10	*E. coli*	410	NDM-5								CTX-M-15				
eco-11	*E. coli*	2,003				TEM-1					CTX-M-14				
eco-12	*E. coli*	2,003				TEM-2					CTX-M-55				
eco-13	*E. coli*	224											DHA-1		
eco-14	*E. coli*	410	NDM-5			TEM-1		OXA-1			CTX-M-55			CMY-2	
eco-16	*E. coli*	676	NDM-1							SHV-12	CTX-M-216				
eco-17	*E. coli*	38							TEM-243		CTX-M-27				
eco-19	*E. coli*	410	NDM-5			TEM-1					CTX-M-14				
eae-1	*K. aerogenes*	14				TEM-1					CTX-M-14			CMY-132	
eae-3	*K. aerogenes*	208				TEM-1				SHV-2	CTX-M-3			CMY-132	
eae-4	*K. aerogenes*	14	NDM-5			TEM-1								CMY-132	
eae-5	*K. aerogenes*	14												CMY-132	
eae-6	*K. aerogenes*	14	NDM-5			TEM-1								CMY-132	
eae-7	*K. aerogenes*	14	NDM-5			TEM-1								CMY-132	
eae-8	*K. aerogenes*	14	NDM-5			TEM-1								CMY-132	
eae-9	*K. aerogenes*	14	NDM-5			TEM-1								CMY-132	
eae-10	*K. aerogenes*	14	NDM-1			TEM-1				SHV-12				CMY-132	
eae-11	*K. aerogenes*	14	NDM-5			TEM-1								CMY-132	
ecl-1	*E. cloacae*	–^*a*^		IMP-8		TEM-1									ACT-2
ecl-2	*E. cloacae*	110	NDM-1			TEM-1				SHV-12					ACT-27
ecl-5	*E. cloacae*	125													ACT-28
ecl-6	*E. cloacae*	270													ACT-84
ecl-10	*E. cloacae*	50		IMP-26		TEM-1							DHA-1		ACT-15
ecl-16	*E. cloacae*	789													
ecl-17	*E. cloacae*	420													ACT-15
ecl-18	*E. cloacae*	114													ACT-69
pvu-1	*P. vulgaris*	–^*a*^													
cfr-1	*C. freundii*	–^*a*^	NDM-1								CTX-M-14			CMY-152	

^*a*^ –, the sequence type (ST) of the strain could not be matched with any known STs in the corresponding MLST database.

**Fig 1:**
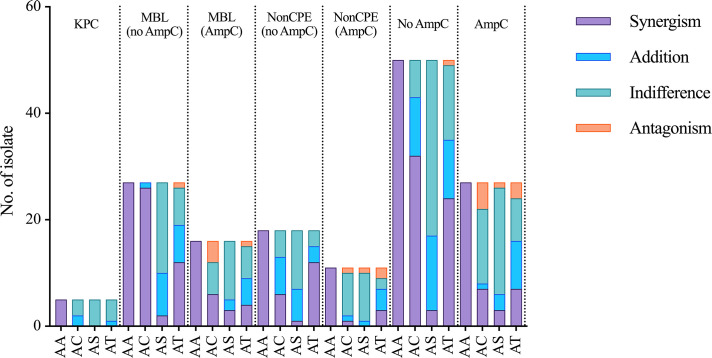
Combined effects of aztreonam with and four β-lactamase inhibitors on CRE isolates. AA, aztreonam combined with avibactam; AC, aztreonam combined with clavulanic acid; AS, aztreonam combined with sulbactam; AT, aztreonam combined with tazobactam; no AmpC, no AmpC enzyme-encoding genes; non-CPE, noncarbapenemase gene-harboring Enterobacteriaceae.

The results of separate analyses of different resistance mechanisms revealed that aztreonam combined with avibactam had a synergistic effect on all the tested isolates. Among the CRE-harboring KPC gene isolates, aztreonam combined with clavulanic acid or tazobactam had an additive effect on 2/5 and 1/5 of the isolates, respectively. Among the CRE isolates that harbored MBL genes but not AmpC enzyme-encoding genes, aztreonam combined with clavulanic acid had a synergistic effect on 26/27 isolates, followed by aztreonam combined with tazobactam, with a synergistic effect on 12/27 isolates. Among the CRE isolates that harbored MBL and AmpC enzyme-encoding genes, aztreonam combined with clavulanic acid had synergistic effects on 6/16 isolates and antagonistic effects on 4/16 isolates. In the noncarbapenemase gene-harboring and non-AmpC enzyme-encoding gene-harboring groups, aztreonam combined with clavulanic acid and tazobactam had synergistic effects on 6/18 isolates and 12/18 isolates, respectively, whereas synergistic effects were observed for these combinations for only 1/11 and 3/11 isolates, respectively, in the AmpC enzyme-encoding gene-harboring group (Fig. 1).

### Antimicrobial susceptibility at different β-lactamase/enzyme inhibitor concentration ratios

**Fig 2:**
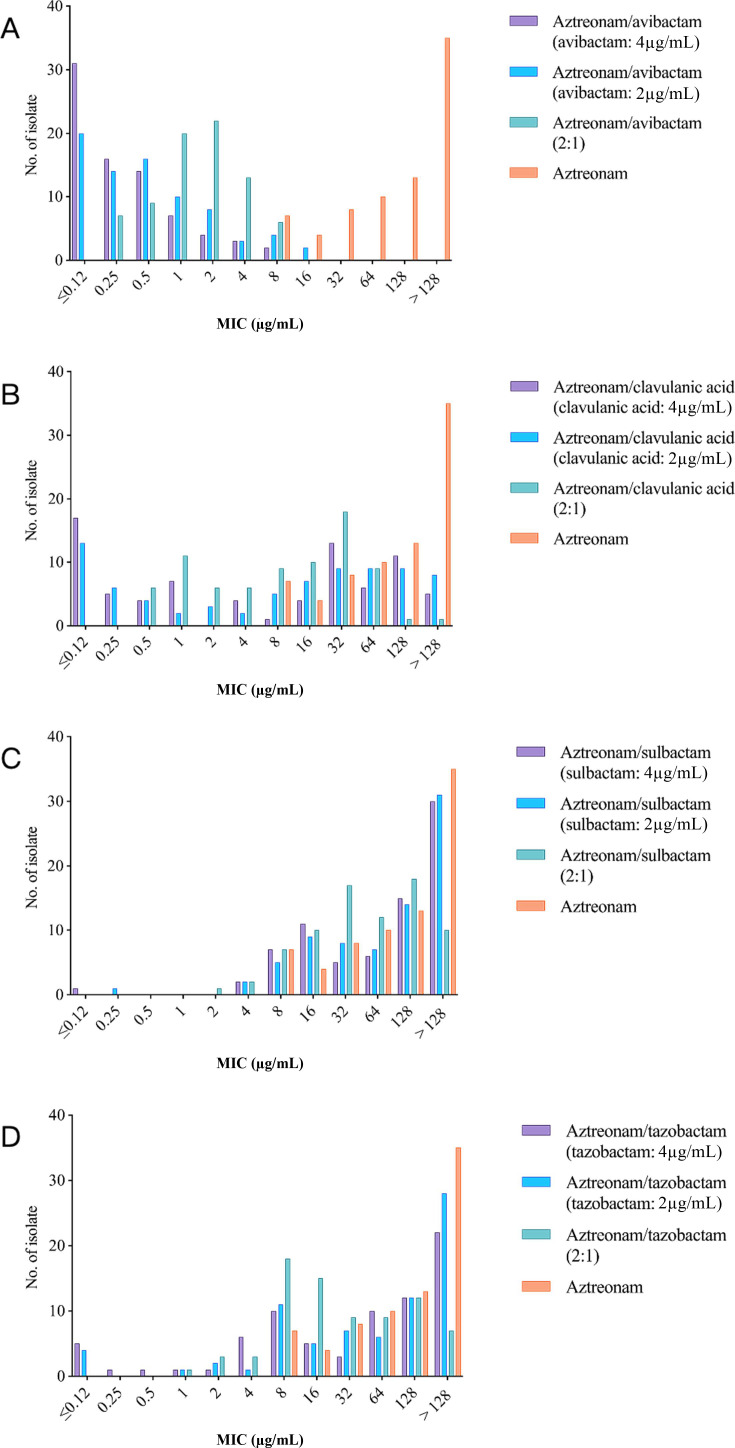
MIC distributions of aztreonam combined with four β-lactamase inhibitors at different concentration ratios for CRE isolates. (**A**) Aztreonam combined with avibactam. (**B**) Aztreonam combined with clavulanic acid. (**C**) Aztreonam combined with sulbactam. (**D**) Aztreonam combined with tazobactam.

To understand the combined effects of different types of aztreonam and different inhibitors, we tested the sensitivity of these strains to drug combinations of different ratios and formulations. Compared with aztreonam alone, aztreonam combined with avibactam had the most marked MIC reduction, followed by aztreonam combined with clavulanic acid and aztreonam combined with tazobactam, but the reduction in the MIC of aztreonam combined with sulbactam was not obvious (Fig. 2). Among the different concentration ratios, the lowest MIC50 and MIC90 values were observed for aztreonam/avibactam (avibactam: 4 µg/mL), aztreonam/clavulanic acid (2:1), aztreonam/sulbactam (2:1), and aztreonam/tazobactam (2:1). In the KPC gene-harboring group and the AmpC enzyme-encoding gene-harboring group (including MBL gene-harboring and noncarbapenemase gene-harboring isolates), the MIC50 and MIC90 values of aztreonam combined with avibactam were much lower than those of aztreonam alone; among the other groups, only the MIC50 of aztreonam/tazobactam (2:1) was only slightly lower than that of either drug alone. In the group that harbored MBL and not AmpC enzyme-encoding genes, the MIC50 and MIC90 values of aztreonam/clavulanic acid (clavulanic acid: 4 µg/mL) were ≤0.12 µg/mL and 1 µg/mL, respectively. These values were similar to those of aztreonam/avibactam (avibactam: 4 µg/mL) and were much lower than those of aztreonam alone (MIC50: 128 µg/mL; MIC90: >128 µg/mL). In the group that did not harbor carbapenemase genes and did not harbor AmpC enzyme-encoding genes, the MIC50 and MIC90 values of aztreonam/clavulanic acid (2:1) were 16 µg/mL and 64 µg/mL, respectively, which were slightly lower than those of aztreonam alone. In the non-AmpC enzyme-encoding gene-harboring group (including the MBL gene-harboring and noncarbapenemase gene-harboring groups), the MIC50 values (1 µg/mL) of aztreonam/clavulanic acid (clavulanic acid: 4 µg/mL) were much lower than the MIC50 values of aztreonam alone (>128 µg/mL), the MIC50 values of aztreonam/tazobactam (2:1) and aztreonam/sulbactam (2:1) were slightly lower than the MIC50 value of aztreonam alone, and the MIC50 values of aztreonam/tazobactam (2:1) were lower than that of aztreonam/sulbactam (2:1; Table 4).

**TABLE 4:** Antimicrobial susceptibility of CRE that harbor different β-lactamase genes to aztreonam combined with four β-lactamase inhibitors at different concentrations

Antibiotic combinations	All (*n* = 77)		KPC (*n* = 5)		MBL (no AmpC; *n* = 27)		MBL (AmpC; *n* = 16)		Non-CPE (no AmpC; *n* = 18)		Non-CPE (AmpC; *n* = 11)		No AmpC (*n* = 50)		AmpC (*n* = 27)	
	MIC50 (µg/mL)	MIC90 (µg/mL)	MIC50 (µg/mL)	MIC90 (µg/mL)	MIC50 (µg/mL)	MIC90 (µg/mL)	MIC50 (µg/mL)	MIC90 (µg/mL)	MIC50 (µg/mL)	MIC90 (µg/mL)	MIC50 (µg/mL)	MIC90 (µg/mL)	MIC50 (µg/mL)	MIC90 (µg/mL)	MIC50 (µg/mL)	MIC90 (µg/mL)
Aztreonam	>128	>128	>128	>128	128	>128	32	>128	>128	>128	128	>128	>128	>128	64	>128
Aztreonam/avibactam (avibactam: 4 µg/mL)	0.25	2	0.5	1	≤0.12	0.5	0.25	1	≤0.12	4	1	4	≤0.12	1	0.5	2
Aztreonam/avibactam (avibactam: 2 µg/mL)	0.5	4	1	2	0.5	1	0.5	2	0.25	8	1	8	0.25	2	0.5	8
Aztreonam/avibactam (2:1)	2	4	4	4	1	2	1	4	2	8	4	8	2	4	2	8
Aztreonam/clavulanic acid (clavulanic acid: 4 µg/mL)	16	128	128	>128	≤0.12	1	32	64	16	>128	128	128	1	128	32	128
Aztreonam/clavulanic acid (clavulanic acid: 2 µg/mL)	16	>128	>128	>128	0.25	8	32	64	32	>128	128	>128	2	>128	64	128
Aztreonam/clavulanic acid (2:1)	16	64	64	>128	1	8	32	32	16	64	32	64	4	64	32	64
Aztreonam/sulbactam (sulbactam: 4 µg/mL)	128	>128	>128	>128	128	>128	16	>128	128	>128	128	>128	128	>128	64	>128
Aztreonam/sulbactam (sulbactam: 2 µg/mL)	128	>128	>128	>128	128	>128	16	>128	128	>128	128	>128	128	>128	32	>128
Aztreonam/sulbactam (2:1)	64	>128	>128	>128	32	>128	32	128	64	>128	64	128	64	>128	32	128
Aztreonam/tazobactam (tazobactam: 4 µg/mL)	64	>128	>128	>128	64	>128	8	>128	64	>128	128	>128	64	>128	16	>128
Aztreonam/tazobactam (tazobactam: 2 µg/mL)	128	>128	>128	>128	128	>128	16	>128	128	>128	128	>128	128	>128	32	>128
Aztreonam/tazobactam (2:1)	16	128	>128	>128	16	128	8	128	16	128	16	128	16	>128	16	128

### Time-kill assay

**Fig 3:**
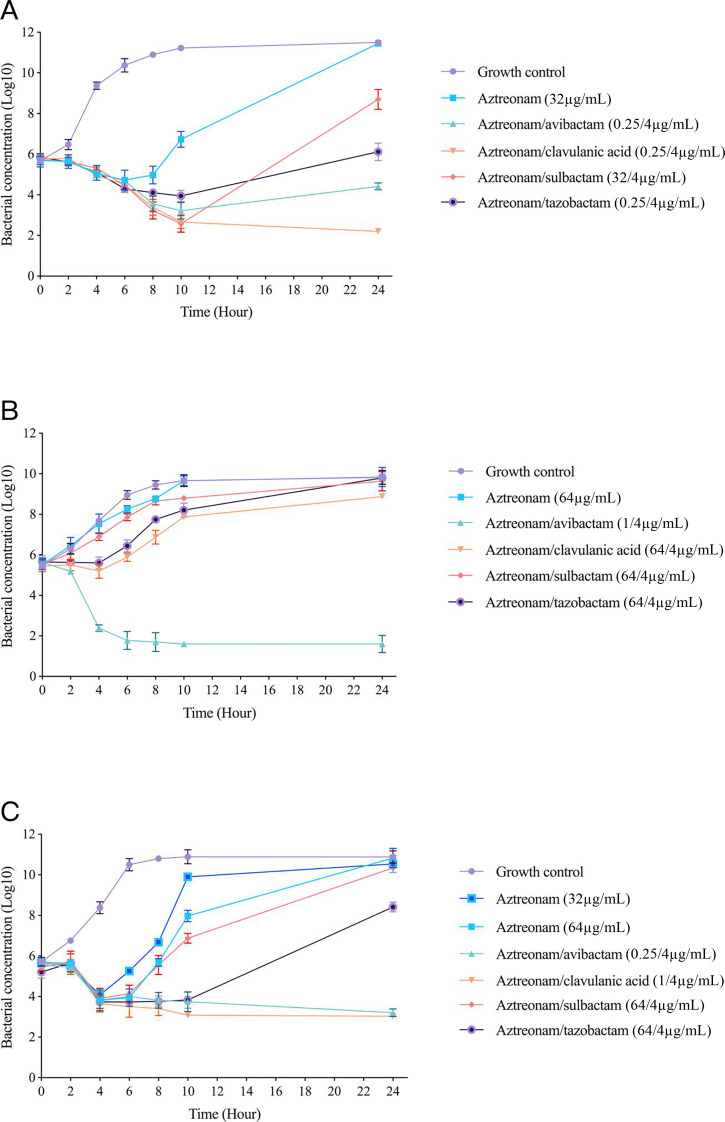
Mean log10 CFU/mL versus time profile for aztreonam (ATM) alone and in combination with 4 µg/mL avibactam, clavulanic acid, sulbactam, or tazobactam against three *K. pneumoniae* strains. Curves represent the average concentrations from triplicate experiments, with error bars indicating the corresponding standard deviation. (**A**) kpn-6 (ST307; IMP-38, TEM-1, SHV-28, and CTX-M-3; ATM alone MIC: 64 µg/mL). (**B**) kpn-12 (ST11; KPC-2, TEM-1, SHV-11, and CTX-M-65; ATM alone MIC > 128 µg/mL). (**C**) kpn-47 (ST273; NDM-5, TEM-1, SHV-11, and CTX-M-3; ATM alone MIC: 128 µg/mL).

According to the test results in Table 4, we selected kpn-6, kpn-12, and kpn-47 from the most detected strains (*K. pneumoniae*) that can represent common ST types (ST307 and ST11) and carry common carbapenemase genes (NDM-5, KPC-2, and IMP-38) for the time‒kill curve test (Fig. 3). The results revealed that kpn-6 harbors the IMP-38 gene, as well as the genes encoding TEM-1 (an NSBL), SHV-28 (an ESBL), and CTX-M-3 (an ESBL), and the MIC value of aztreonam alone was 64 µg/mL. Compared with 32 µg/mL aztreonam alone, 0.25 µg/mL aztreonam combined with 4 µg/mL clavulanic acid clearly reduced the bacterial concentration (by 10^3^); 0.25 µg/mL aztreonam combined with avibactam or tazobactam reduced the bacterial concentration slightly less than clavulanic acid did, and the bactericidal effect of 32 µg/mL aztreonam combined with 4 µg/mL sulbactam at 10 h was similar to that of aztreonam combined with clavulanic acid, but the bacterial concentration at 24 h was greater than the initial bacterial concentration at 0 h.

For isolate kpn-12, which harbors the KPC-2, TEM-1 (an NSBL), SHV-11 (an ESBL), and CTX-M-65 (an ESBL) genes, the MIC value of aztreonam alone was >128 µg/mL, whereas 1 µg/mL aztreonam combined with 4 µg/mL avibactam reduced the bacterial concentration by 10^3^. Compared with that in the group treated with 64 µg/mL aztreonam alone, the bacterial concentration in the other groups was slightly lower after the addition of 4 µg/mL sulbactam, tazobactam, or clavulanic acid; among these agents, clavulanic acid reduced the bacterial concentration more than tazobactam and sulbactam did.

For kpn-47, which harbors NDM-5, TEM-1 (an NSBL), SHV-11 (an ESBL), and CTX-M-3 (an ESBL), the MIC value of aztreonam alone was 128 µg/mL; compared with 64 µg/mL aztreonam alone, the addition of 4 µg/mL sulbactam did not clearly reduce the bacterial concentration, whereas the addition of 4 µg/mL tazobactam reduced the bacterial concentration by 10^3^. Aztreonam (0.25 µg/ml) combined with 4 µg/mL avibactam and 1 µg/mL aztreonam combined with 4 µg/mL clavulanic acid could achieve good synergistic bactericidal effects within 24 h.

## DISCUSSION

CRE’s multidrug resistance restricts the antibiotic arsenal available for treatment. Furthermore, the toxic side effects of many antibiotics disproportionately impact pediatric patients, leaving fewer viable therapeutic options for children than for adults. Our results are similar to those of other studies (5, 6). The aztreonam-resistant CRE isolates detected in children in Shenzhen presented high resistance rates and high MICs for the tested antimicrobial drugs, except for amikacin, polymyxin B, and tigecycline. Among the evaluated resistance factors, MBL had a high detection rate; this was consistent with the high resistance rate to ceftazidime/avibactam and similar to the findings of other studies on children in China (6, 16).

The results of this study showed that aztreonam-resistant CRE isolates harboring the MBL gene typically also carry other β-lactamase genes, among which various β-lactamases, in addition to MBL, can hydrolyze aztreonam and cause resistance (34, 35). Avibactam can inhibit NSBLs, ESBLs, and AmpC enzymes but not MBLs (8, 36). Therefore, aztreonam combined with avibactam has good synergistic antibacterial effects against aztreonam-resistant CRE isolates that harbor multiple β-lactamase genes at the same time, whereas clavulanic acid, sulbactam, and tazobactam have inhibitory effects on only NSBL and ESBL, and their inhibitory enzyme effects also vary with the enzyme category (37). The results of this study revealed that, in CRE isolates with MBL-encoding genes but not AmpC-encoding genes, aztreonam combined with clavulanic acid presented synergistic antibacterial effects similar to those of aztreonam combined with avibactam, whereas the synergistic antibacterial effect of aztreonam combined with tazobactam was lower than that of aztreonam combined with clavulanic acid; however, the effect of aztreonam combined with sulbactam was mostly indifferent. While the checkerboard and microbroth dilution methods read at 16–20 h showed indifferent effects on the effects of the aztreonam/sulbactam combination, time‒kill curve assays demonstrated clear synergistic bactericidal activity against some CRE isolates within 10 h. Furthermore, given that most β-lactam antibiotics are administered every 8 h in clinical practice, the actual clinical use of aztreonam and sulbactam combination regimens requires further study. Since clavulanic acid, sulbactam, and tazobactam all fail to inhibit AmpC enzymes well and clavulanic acid can even induce high expression of some AmpC enzymes (35), the results of this study showed that, for CRE isolates harboring both MBL and AmpC enzyme-encoding genes, the antagonistic effect of aztreonam combined with clavulanic acid was stronger than that of the other three treatments. Among the CRE-harboring KPC genes, aztreonam combined with clavulanic acid had an additive effect, which may be due to the weak inhibitory effect of clavulanic acid on KPC (38–40). Since the detection rate of ESBL genes in *E. coli* and *K. pneumoniae* is usually greater than that of AmpC genes, whereas the opposite is true for *K. aerogenes* and the *E. cloacae* complex, the synergistic effect of aztreonam combined with clavulanic acid on aztreonam-resistant *E. coli* or *K. pneumoniae* is stronger than that on *K. aerogenes* or the *E. cloacae* complex; in contrast, the antagonistic effect is weaker than that on *K. aerogenes* or the *E. cloacae* complex.

The concentration ratio of β-lactams to β-lactamase inhibitors is also an important indicator for antimicrobial susceptibility testing in clinical microbiology laboratories and for the selection of clinical anti-infection treatments. For example, the ratio of amoxicillin/clavulanic acid and ampicillin/sulbactam used in the CLSI is 2:1, whereas the concentration of β-lactamase inhibitors in piperacillin/tazobactam and ceftazidime/avibactam is fixed at 4 µg/mL (7). In clinical treatment, the dosage and dosage form are more complicated due to the consideration of factors such as the MIC value and PK/PD are considered. The results of this study revealed that among all the tested isolates, the MIC50 and MIC90 values of aztreonam combined with avibactam were lowest when the avibactam concentration was fixed at 4 µg/mL, whereas those of the other three treatments were the lowest when this ratio was 2:1, possibly because avibactam is a nonsuicidal β-lactamase inhibitor (36), which is different from the other three β-lactamase inhibitors. Moreover, when MBL genes are harbored and AmpC enzymes are not harbored, the MIC50 and MIC90 values of aztreonam combined with clavulanic acid at a fixed concentration of 4 µg/mL are similar to those of aztreonam combined with avibactam at a fixed concentration of 4 µg/mL, which provides a reference for clinical anti-infection treatment based on PK/PD in the future.

The IDSA 2024 guidance recommends the use of aztreonam/avibactam or cefiderocol for the treatment of infections other than urinary tract infections caused by MBL-producing CRE (14). However, our results showed that the MIC of the β-lactamase inhibitor combination of aztreonam and clavulanic acid was very low or that it had an *in vitro* synergistic effect against some CRE strains harboring MBL genes but not AmpC enzyme-encoding genes. These findings suggest that for infections caused by CRE strains with these antimicrobial resistance mechanisms, aztreonam combined with clavulanic acid may be a potential alternative treatment option to aztreonam/avibactam. Since whole-genome sequencing was used in this study to detect β-lactamase-encoding genes, enzyme expression or activity was not experimentally confirmed. CLSI currently has no recommended phenotypic detection method for AmpC enzymes (7); the phenotypic detection methods for AmpC enzymes reported in previous studies usually use cefoxitin as an indicator drug (41). However, since multiple carbapenemases can hydrolyze cefoxitin (42), the commonly used phenotypic detection methods for CRE cannot currently identify whether it produces AmpC enzymes. It can only be preliminarily inferred through *in vitro* susceptibility tests or combined antimicrobial susceptibility testing whether the treatment regimen of aztreonam combined with clavulanic acid may be effective. Notably, this study provides the results of only single-center *in vitro* tests. Although the effectiveness of this treatment has been reported in a small number of cases (20), it is still necessary to consider the severity of the patient’s condition and the PK/PD of aztreonam and β-lactamase inhibitors (43) and confirm the treatment selection through further animal tests and clinical trials before using this approach in clinical applications. If proven effective in clinical practice, its advantage lies in reducing the economic burden on patients and simultaneously reducing the use of aztreonam/avibactam to delay the emergence of aztreonam/avibactam resistance. In addition, owing to the widespread dissemination of the IncX3 plasmid carrying NDM-5 (44), it may be worthwhile commenting on the activity of the combination regimens among those specific isolates harboring NDM-5. However, the clinical applicability of aztreonam/clavulanic acid may be limited by regional drug availability, as intravenous clavulanic acid formulations are not accessible in some countries (e.g., the United States). Notably, although carbapenemase production is the main resistance mechanism of CRE, other resistance mechanisms (porin changes, efflux pumps, penicillin-binding protein mutations, etc.) can lead to resistance of *Enterobacterales* to carbapenems (8). The antibacterial activity of aztreonam combined with clavulanic acid against CRE with these resistance mechanisms still needs further study.

In addition, continuous monitoring of the resistance of *Enterobacterales* and *S. maltophilia* to aztreonam/avibactam is very important. At present, *in vitro*-induced resistance and clinical isolation of isolates of *Enterobacterales* and *S. maltophilia* resistant to aztreonam/avibactam have been reported (19, 45, 46). With the widespread clinical application of aztreonam/avibactam in many countries and regions worldwide, the detection rate of resistant bacteria and reports on resistance mechanisms are expected to gradually increase.

Some results of this study are similar to those of previous studies (20, 22–25), but the test isolates were isolated from children in Shenzhen, the number of test isolates was greater, and the standard checkerboard dilution method and time‒kill curve method were used to verify the synergistic effects. In addition, our study revealed that the antagonistic effects of aztreonam combined with clavulanic acid on some isolates may be due to the production of AmpC enzymes. Furthermore, this study was conducted only *in vitro*. We look forward to large-scale clinical trials in the future to confirm the results.

### Conclusions

In conclusion, a high proportion of aztreonam-resistant CRE isolates from children in Shenzhen harbored MBL and other β-lactamase genes at the same time. Aztreonam/avibactam had good antibacterial activity against all the tested isolates. Aztreonam/clavulanic acid can be used as a more economical combination treatment than aztreonam/avibactam for isolates that harbor MBL genes but not AmpC enzyme-encoding genes, but it is not suitable for isolates that harbor AmpC enzyme-encodingitro Combined Inhibitory Activities of β-Lactam Antibi genes or the KPC gene.

## Data Availability

The WGS genome sequences data of 77 aztreonam-resistant CREs were deposited at SRA under Bioproject with accession number PRJNA1381745.

## SUPPLEMENTAL MATERIAL

Table S1 spectrum.01408-25-s0001.xlsx Combined antimicrobial susceptibility testing results of 77 aztreonam-resistant CRE isolates.

ASM does not own the copyrights to Supplemental Material that may be linked to, or accessed through, an article. The authors have granted ASM a non-exclusive, world-wide license to publish the Supplemental Material files. Please contact the corresponding author directly for reuse.
