# Erratum for Meng et al., “Comparative analysis of the effects of aztreonam combined with four β-lactamase inhibitors against carbapenem-resistant *Enterobacterales*”

**DOI:** 10.1128/spectrum.00790-26

**Published:** 2026-08-25

**Authors:** Qing Meng, Chao Xu, Wujiao Li, Kunling Shen, Xiuhui Huang, Xiaoying Fu, Lintao Zhou, Yunsheng Chen, Fupin Hu, Heping Wang

## ERRATUM

Volume 14, no. 3, e01408-25, 2026, https://doi.org/10.1128/spectrum.01408-25.

Acknowledgments section and Funding table: The following funding information should be added:

Funder: Guangdong Provincial High-level Hospital Construction Special Fund

Grant(s): YNKT2021

Author(s): Kunling Shen

